# Glioblastoma and its treatment are associated with extensive accelerated brain aging

**DOI:** 10.1111/acel.14066

**Published:** 2024-01-17

**Authors:** Anna P. Ainslie, Myrthe Klaver, Daniëlle C. Voshart, Emma Gerrits, Wilfred F. A. den Dunnen, Bart J. L. Eggen, Steven Bergink, Lara Barazzuol

**Affiliations:** ^1^ Department of Radiation Oncology University Medical Center Groningen, University of Groningen Groningen The Netherlands; ^2^ Department of Biomedical Sciences of Cells and Systems University Medical Center Groningen, University of Groningen Groningen The Netherlands; ^3^ European Research Institute for the Biology of Ageing University Medical Center Groningen, University of Groningen Groningen The Netherlands; ^4^ Department of Pathology and Medical Biology University Medical Center Groningen, University of Groningen Groningen The Netherlands; ^5^ University College Groningen, University of Groningen Groningen The Netherlands

**Keywords:** aging hallmarks, cancer treatment side effects, cognitive decline, glioblastoma, neurodegeneration

## Abstract

Progressive neurocognitive dysfunction is the leading cause of a reduced quality of life in patients with primary brain tumors. Understanding how the human brain responds to cancer and its treatment is essential to improve the associated cognitive sequelae. In this study, we performed integrated transcriptomic and tissue analysis on postmortem normal‐appearing non‐tumor brain tissue from glioblastoma (GBM) patients that had received cancer treatments, region‐matched brain tissue from unaffected control individuals and Alzheimer's disease (AD) patients. We show that normal‐appearing non‐tumor brain regions of patients with GBM display hallmarks of accelerated aging, in particular mitochondrial dysfunction, inflammation, and proteostasis deregulation. The extent and spatial pattern of this response decreased with distance from the tumor. Gene set enrichment analyses and a direct comparative analysis with an independent cohort of brain tissue samples from AD patients revealed a significant overlap in differentially expressed genes and a similar biological aging trajectory. Additionally, these responses were validated at the protein level showing the presence of increased lysosomal lipofuscin, phosphorylated microtubule‐associated protein Tau, and oxidative DNA damage in normal‐appearing brain areas of GBM patients. Overall, our data show that the brain of GBM patients undergoes accelerated aging and shared AD‐like features, providing the basis for novel or repurposed therapeutic targets for managing brain tumor‐related side effects.

## Graphical abstract

In this study, we performed integrated transcriptomic and tissue analysis comparing postmortem non‐tumor brain tissue from glioblastoma (GBM) patients that had received cancer treatments, with region‐matched brain tissue from unaffected control individuals and Alzheimer's disease patients. We show that normal‐appearing non‐tumor brain regions of patients with GBM display hallmarks of accelerated aging, in particular mitochondrial dysfunction, inflammation, and proteostasis deregulation.

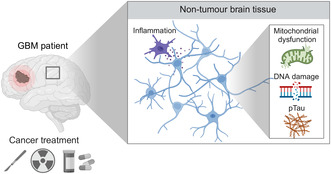

**Abbreviations**
ApoE: apolipoprotein E
AD: Alzheimer's disease
BDNF: brain‐derived neurotrophic factor
COMT: catechol‐O‐methyltransferase
DEGs: differentially expressed genes
GBM: glioblastoma
NDs: neurodegenerative diseases
PCA: Principle Component Analysis
PMI: post mortem interval
SNPs: single nucleotide polymorphisms

## 1 INTRODUCTION

Recent advances in brain tumor treatment have led to an increase in the proportion of long‐term survivors depending on tumor subtypes and pediatric versus adult patients (Ostrom et al., 2021, 2022). In adult cases, the median overall survival ranges between 14.6 months for grade IV glioblastoma (GBM) and 13.8 years for lower grade II glioma (Wefel et al., 2016), while in the pediatric population the 5‐year survival rate has increased to over 75% (Gondi et al., 2016). Notably, the prognosis of GBM patients worsens with age (Kim et al., 2021; Ladomersky et al., 2020; Lowry et al., 1998). Nearly all patients with primary brain tumors develop debilitating neurocognitive dysfunction, resulting in a reduced quality of life, and educational and occupational attainment (Al Dahhan et al., 2022; Liu et al., 2009; Lustberg et al., 2023). Importantly, this impairment in neurocognitive function is irreversible and progressive in nature, in some cases developing long after completion of treatment (Al Dahhan et al., 2022; Makale et al., 2017). It affects various neurocognitive domains, in particular memory, processing speed, and executive function, resulting in dementia in 5% of cases (Ajithkumar et al., 2017; DeAngelis et al., 1989).

Many factors play a role in the development of neurocognitive dysfunction, including tumor type, size and location, and type of cancer treatment, which often involves a combination of surgery, radiotherapy, and chemotherapy (Wefel et al., 2016). In particular, radiotherapy and chemotherapy have extensively been shown to trigger progressive brain atrophy and affect cognitive outcome (Dietrich, 2010; Hoffmann et al., 2018). Additionally, genome‐wide association studies have reported a number of single nucleotide polymorphisms (SNPs) that are associated with worse neurocognitive outcome in patients with adult brain tumors after treatment with chemotherapy and radiotherapy (Siegel et al., 2019; Wefel et al., 2016). Interestingly, several of these SNPs are in genes implicated in Alzheimer's disease (AD), such as apolipoprotein E (*ApoE*), catechol‐O‐methyltransferase (*COMT*), and brain‐derived neurotrophic factor (*BDNF*) (Wefel et al., 2016).

Despite the major impact of neurocognitive dysfunction, the mechanisms mediating this cognitive decline in patients with brain tumors remain largely unknown. Most research on gliomas have focused on tumor samples and neuronal activity‐regulated cancer growth (Venkataramani et al., 2019; Venkatesh et al., 2015, 2019). Here, we investigated how the healthy human brain responds to cancer and its treatment by performing comparative transcriptional profiling of multiple postmortem brain samples from patients with GBM. We found that normal‐appearing non‐tumor brain regions from GBM patients display extensive mis‐regulation of genes involved in inflammation and mitochondrial function. Gene set enrichment analyses and a direct comparative transcriptomic analysis with an independent cohort of brain samples from AD patients revealed a significant overlap with AD. Furthermore, histological and protein analyses showed an increase in oxidative damage and other hallmarks of aging in normal‐appearing brain regions from GBM patients. Overall, these data indicate that the brain of GBM patients undergoes accelerated aging with a similar biological trajectory to AD.

## 2 MATERIALS AND METHODS

### 2.1 Study design

The collection of postmortem samples from deceased individuals and associated distribution for research was approved by the NIH NeuroBioBank (provider institution) and the University Medical Center Groningen (UMCG; recipient institution) under request IDs 1116, 1834, and 2463. Although the collection of biospecimens from deceased individuals is not legally classified as human subjects research (under 45 CFR Part 46), donor recruitment sites typically obtained written or telephonic authorization and informed consent. Symptom categories in Figure S1 were adapted from Mekkes et al. (2022).

Post mortem samples from unaffected control individuals, GBM patients, and AD patients (Braak stages 4–6) were selected based on age (between 44 and 62 years old) and sex (similar distribution of male and female). From each GBM patient, we collected two normal‐appearing brain tissue samples (NA‐GBM) that were in proximity to the tumor (subsequently denoted as “Near #1” and “Near #2”), and one normal‐appearing brain tissue sample further from the tumor (subsequently denoted as “Far”) (Data S1). Unaffected control samples were selected to be accordingly region matched to the NA‐GBM samples. AD patient samples were region‐matched to GBM Near #1 regions. Control samples were confirmed to be neurotypical by the NIH NeuroBioBank. Additionally, a neuropathologist confirmed lack of brain tumor cells in our NA‐GBM samples, with the exception of samples from one GBM patient (GBM_4, regions Near #1 and Near #2) that contain some infiltrating tumor cells.

**TABLE 1:** Postmortem brain tissue samples used in this study.

Patient	Age (years)	Sex	Sequenced	Histological analysis	Western blot	PMI	Overall survival	Radiotherapy	Chemotherapy
Control_1	54	F	Yes	Yes	Yes	6	NA	NA	NA
Control_2	60	M	Yes	Yes	Yes	37	NA	NA	NA
Control_3	54	M	Yes	No	No	9	NA	NA	NA
Control_4	50	F	Yes	No	Yes	15	NA	NA	NA
Control_5	49	F	Yes	Yes	Yes	26	NA	NA	NA
Control_6	46	F	No	Yes	No	18	NA	NA	NA
Control_7	54	F	No	Yes	No	27	NA	NA	NA
Control_8	63	F	No	Yes	No	25	NA	NA	NA
GBM_1	62	M	Yes	No	No	7	1–2 years	Yes	Yes, Temozolomide, Irinotecan
GBM_2	57	F	Yes	No	Yes	8	<1 year	Yes	Yes, Temozolomide
GBM_3	55	F	Yes	Yes	Yes	21	2–3 years	Yes	Not described in medical records
GBM_4	60	F	Yes	No	Yes	21	3–6 months	Yes	Yes, Temozolomide
GBM_5	49	F	Yes	Yes	Yes	3	1–2 years	Yes	Yes, Temozolomide
GBM_6	59	M	No	Yes	No	9	<1 year	Not described in medical records	Not described in medical records
GBM_7	55	F	No	Yes	No	18	Not described in medical records	Yes	Yes, Irinotecan
GBM_8	61	F	No	Yes	No	4	4 months	Yes	Yes, Temozolomide
GBM_9	47	F	No	Yes	No	71	1 year	Yes	Yes, Temozolomide
AD_1	60	F	Yes	No	Yes	15	NA	NA	NA
AD_2	44	M	Yes	No	Yes	14	NA	NA	NA
AD_3	59	M	Yes	No	No	13	NA	NA	NA
AD_4	56	M	Yes	Yes	Yes	4	NA	NA	NA
AD_5	59	F	Yes	No	No	19	NA	NA	NA
AD_6	56	F	No	Yes	No	4	NA	NA	NA
AD_7	59	F	No	Yes	No	6	NA	NA	NA

*Note*: Table includes age at time of death, postmortem interval in hours, sex, whether they are included in the RNA‐sequencing, immunohistochemistry, and western blot analyses. Patient information of the GBM patient samples used in this study, including their survival from diagnosis, whether they have radiotherapy and chemotherapy. Further patient information is available in Data S1.

Abbreviations: M, male; F, female.

### 2.2 RNA quality and sequencing

RNA isolation was performed on 22 unaffected control, 25 NA‐GBM, and 11 AD brain tissue samples. A total of 35 brain tissue samples from five unaffected control individuals, five NA‐GBM, and five AD patient donors were selected for RNA‐sequencing (see Section 2.3). Approximately 40 mg of frozen brain tissue was processed using Qiagen RNA Lipid Tissue Kit. Quality of the RNA was determined using TapeStation, only samples with a RIN >4.5 were included in the experiment. 70 ng of sample RNA was used for library preparation with the Lexogen QuantSeq 3′ mRNA‐Seq Library Prep Kit (FWD). cDNA libraries were pooled equimolarly and approximately 5 M reads per sample were sequenced on a NextSeq 500 at the sequencing facility in the UMCG.

### 2.3 Transcriptomic analysis

Data preprocessing was performed with the Lexogen QuantSeq 2.3.1 FWD UMI pipeline. The gene count files were imported into R. The “boxplot” function was used to check the read count per million read distributions in each sample. Samples with a consistent median were selected for further analysis (median = 3 ± 0.25). Principle component analysis (PCA) was performed on normalized read counts and plots were generated in R using “ggplot2.” PCA plots were used to check for clustering according to postmortem interval (PMI), age, or brain region. Based on these plots, samples were further narrowed down to those with PMI < 37 h. No clustering was found according to age, sex, PMI, or brain region (Figures S2a–d, S4c–e). This resulted in a total of 15 samples from five unaffected control individuals (three samples per patient, region matched to the GBM samples), 15 samples from five GBM patients (three samples per patient, two near the tumor, and one far from the tumor), and five AD samples (one sample per patient) for final DEG analysis. DEG analysis was performed using “edgeR” (Robinson et al., 2010). Samples denoted as Near #1 were sequenced separately to Near #2 and Far. Batch effect correction was performed in R using function “removeBatchEffect” in order to analyze Near #1, Near #2, and Far together. Volcano plots and heat maps were generated using the CRAN package “ggplot2.”

### 2.4 GO analysis and enrichment analysis

**FIGURE 1:**
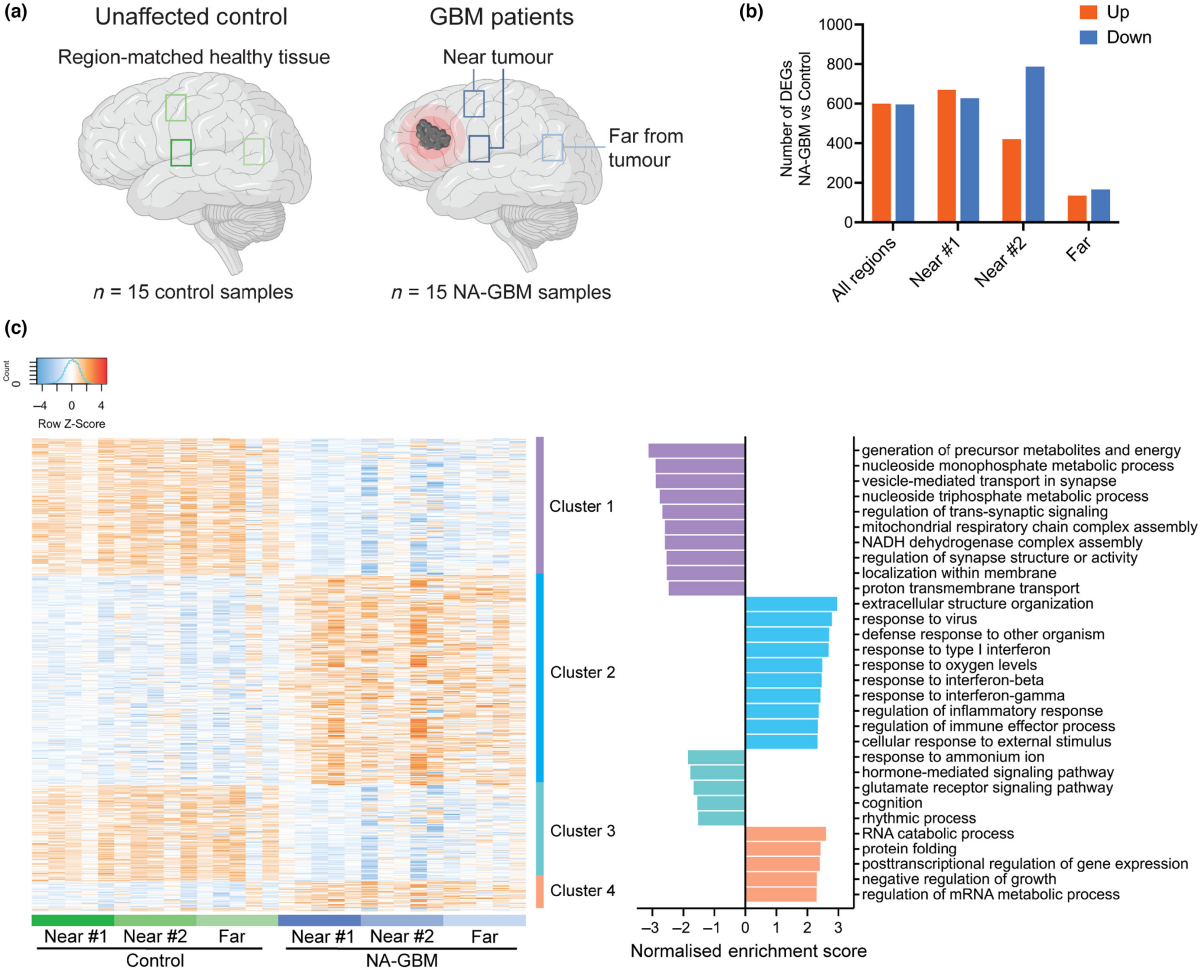
Transcriptomic analysis of normal‐appearing GBM patient brain tissue. (a) Normal‐appearing GBM (NA‐GBM) patient brain tissue samples were analyzed and compared to region‐matched healthy tissue from unaffected control individuals. (b) Number of DEGs when comparing two brain regions near the tumor and one region far from the tumor in five GBM patients, to region‐matched samples derived from five unaffected control individuals (Data S1, S2) (fold change >1.5 and FDR <0.05). (c) Heat map of DEGs logCPM when comparing all NA‐GBM patient samples (two regions near the tumor and one region far from the tumor) to region‐matched unaffected controls (Data S1, S2) (fold change >1.5 and FDR <0.05, *n* = 5 GBM patients and *n* = 5 control individuals). GO analysis (Biological Processes) of individual clusters using WebGestalt (Liao et al., 2019), normalized enrichment is plotted on the right.

**FIGURE 2:**
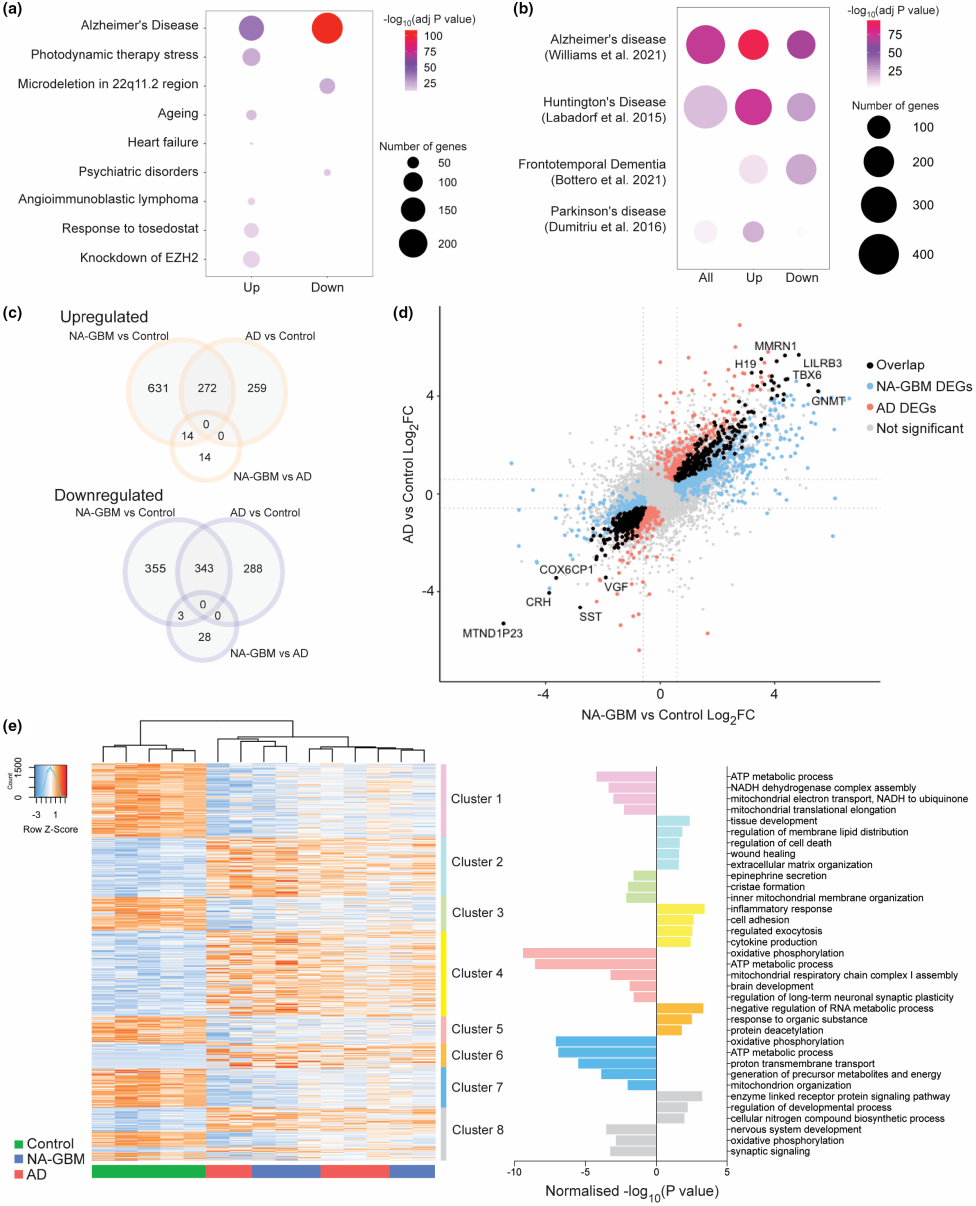
Overlapping gene expression patterns in normal‐appearing GBM and AD patient brain tissue. (a) Results of enrichment analysis, comparing the NA‐GBM (Near #1) versus unaffected control DEG list to the transcriptomic data from the “Chemical and genetic perturbations” data set from the Molecular Signatures Database (MSigDB). The top hits are plotted for the upregulated DEGs and the downregulated DEGs (−log_10_(adjusted *p* value) > 12). Scale bar indicates −log_10_(adjusted *p* value) and dot size represents the number of overlapping genes. (b) Manual comparison of NA‐GBM (Near #1) versus unaffected control DEG list to DEGs from selected neurodegenerative disease transcriptomic studies (Bottero et al., 2021; Dumitriu et al., 2016; Labadorf et al., 2015; Williams et al., 2021). Scale bar indicates −log_10_(adjusted *p* value) and dot size represents the number of overlapping genes. (c) Venn diagram of overlapping upregulated and downregulated DEGs between NA‐GBM (Near #1) and versus unaffected control, AD versus unaffected control and NA‐GBM (Near #1) versus AD data sets, determined on the basis of fold change >1.5 and FDR <0.05 (*n* = 5 GBM patients, *n* = 5 Control individuals, *n* = 5 AD patients). (d) Four‐way plot showing 615 overlapping DEGs between NA‐GBM (Near #1) versus unaffected control and AD versus unaffected control data sets, determined on the basis of fold change >1.5 and FDR <0.05. (e) Clustered heat map of DEGs when comparing NA‐GBM (Near #1) patient samples and AD patient samples together to unaffected controls (fold change >1.5 and FDR <0.05). GO analysis (Biological Processes) of individual clusters using g:Profiler, normalized −log_10_(*p* value) is plotted on the right (Data S1, S2).

GO analysis of the clusters in Figure 1c and GO analysis of all DEGs were performed using WebGestalt (WEB‐based GEne SeT AnaLysis Toolkit, RRID:SCR_006786) (Liao et al., 2019). GO analysis of DEG heat map clusters in Figure 2e was performed using G profiler (Raudvere et al., 2019). Enrichment analysis (Subramanian et al., 2005) of all NA‐GBM versus unaffected control DEGs comparing the data set to the “Chemical and Genetic perturbations” data (Liberzon et al., 2011) was performed by using GSEA software (Mootha et al., 2003; Subramanian et al., 2005), selecting transcriptomic data sets from the top hits for comparison. Enrichment analysis of NA‐GBM Near #1 versus unaffected control DEGs comparing the data set to the “Chemical and Genetic perturbations” data set (Liberzon et al., 2011) was performed using iDEP93 (Ge et al., 2018), selecting transcriptomic data sets from the top hits for comparison. PGSEA comparisons against the Jensen disease database were also calculated using iDEP93 (Ge et al., 2018; Grissa et al., 2022; Pletscher‐Frankild et al., 2015). The number of genes that overlap with transcriptomic data of other neurodegenerative diseases was calculated using the “match” function in R. *p* values were calculated using a hypergeometric distribution test, using the “phyper” function in R. Cell deconvolution analysis was performed using the CIBERSORTx analytical tool (Newman et al., 2019).

### 2.5 Immunofluorescence staining and imaging

Formalin‐fixed and paraffin‐embedded brain tissues from six unaffected control individuals, six GBM patient donors, and three AD patient donors were provided by the NIH NeuroBiobank. Tissue was cut into 5‐μm thick slices and collected on TOMO microscope slides.

The staining procedure for lipofuscin was as follows: paraffin‐embedded tissue sections were de‐paraffinized in xylene and ethanol, then rinsed in demiH_2_O. Slides were washed 3× with PBS, and incubated with DAPI for 10 min. Slides were washed again in PBS 3 × 5 min, and mounted with Faramount Aqueous Mounting Medium.

The staining procedure for 8‐Oxoguanine (8‐oxoG) was as follows: paraffin‐embedded tissue sections were de‐paraffinized in xylene and ethanol, then rinsed in demiH2O. Antigen retrieval was performed on samples by boiling the sections for 3.5 min in HistoVT One (cat# 06380‐05; Nacalai tesque). When the samples were cooled down, they were rinsed in demiH_2_O and incubated with Sudan Black (0.5% in 70% EtOH) for 5 min. After this, the samples were quickly dipped in 70% EtOH, washed 3 × 5 min in demiH_2_O and blocked in PBS+ (PBS + 0.3% Triton) with 2% donkey serum and 2% bovine serum albumin for an hour at room temperature. The samples were then incubated with mouse anti‐8‐Oxoguanine Antibody (cat# MAB3560; Sigma‐Aldrich, RRID:AB_94925, 1:200) primary antibody in PBS+ with 2% donkey serum overnight at 4°C. After washing 3 × 5 min at room temperature in PBS, the sections were incubated for an hour at room temperature with the secondary antibody Alexa Fluor 594 (cat#A‐21203; Invitrogen, RRID:AB_141633, donkey anti‐mouse) in PBS. The samples were washed 3 × 5 min in PBS after which nuclear staining was performed using DAPI for 12 min. The sections were washed for 8 min in PBS, quickly rinsed and mounted using DAKO mounting solution.

### 2.6 Immunohistochemistry

Formalin‐fixed and paraffin‐embedded brain tissues from six unaffected control individuals, six GBM patient donors, and three AD patient donors were provided by the NIH NeuroBiobank. Tissue was cut into 5‐μm thick slices and collected on TOMO microscope slides. The staining procedure for p‐Tau was as follows: paraffin‐embedded tissue sections were de‐paraffinized in xylene and ethanol, then rinsed 1x in demiH_2_O. Antigen retrieval was performed using Histo VT One. Slides were washed 1x in PBS. Peroxidase incubation was performed with 0.5% H_2_O_2_ in PBS for 30 min at room temperature in the dark. Slides were then washed in PBS 3 × 5 min. The slides were blocked with blocking buffer (4% rabbit serum, 1% BSA and 0.1% Triton) for 1 h at room temperature. The slides were incubated with the primary antibody diluted in blocking buffer (p‐Tau (Ser202, Thr205) monoclonal antibody (AT8) cat# MN1020, RRID:AB_223647, used at 1:2000) overnight at 4°C. Slides were then washed in PBS 3 × 5 min and incubated for 1 h with the secondary antibody (biotinylated rabbit anti‐mouse, RRID:AB_2687571, used at 1:300) diluted in blocking buffer at room temperature. The slides were incubated in ABC solution (following the Vectastain elite ABC kit, cat# PK‐6100) for 30 min at room temperature. Slides were then washed in PBS 3 × 5 min. DAB solution was added under a stereoscope, and time elapsed until visible staining occurred in a positive (AD) sample was timed. The same timing was then used for the other samples. The reaction was stopped with demiH_2_O, and the sections were incubated in hematoxylin, then washed with demiH_2_O for 10 min. The sections were dehydrated in an ethanol gradient, mounted with Eukit and dried for 1–2 days.

The staining procedure for amyloid‐β was as follows: paraffin‐embedded tissue sections were de‐paraffinized in xylene and ethanol, then rinsed 1× in demiH_2_O. Antigen retrieval was performed using citric acid and sodium citrate at pH = 6. Slides were washed 3 × 5 min in PBS, then incubated for 3 min at room temperature with formic acid at room temperature. Slides were washed 3 × 5 min in PBS. Blocking solution was prepared (1% donkey serum, 1% BSA in PBS). The slides were incubated with the primary antibody diluted in blocking buffer (β‐Amyloid Antibody Cell Signaling #2454; rabbit, RRID:AB_2056585, used at 1:500) overnight at 4°C. Slides were then washed in PBS 3 × 5 min and incubated for 1 h with the secondary antibody (biotinylated donkey anti‐rabbit, RRID:AB_2340593, used at 1:400) diluted in blocking buffer. The slides were incubated in ABC solution (following the Vectastain elite ABC kit, cat# PK‐6100) for 30 min at room temperature. Slides were then washed in PBS 3 × 5 min. DAB solution was added for 3:30 min at room temperature. The reaction was stopped with demiH_2_O, and the sections were incubated in hematoxylin, then washed with demiH_2_O for 10 min. The sections were dehydrated in an ethanol gradient, mounted with Eukit and dried for 1–2 days.

### 2.7 Image quantification and analysis

The autofluorescence of lipofuscin was imaged using a Leica DM6B microscope. Snapshots were made of each sample on a representative gray‐matter area of 619.57 μm × 464.68 μm with a 20× magnification. Lipofuscin puncta were automatically quantified using an ImageJ (RRID:SCR_002285) macro:

{run(“Enhance Contrast”, “saturated = 0.35″);

run(“Apply LUT”);

run(“Auto Threshold”, “method = Otsu white”);

run(“Gaussian Blur…”, “sigma = 2”);

setOption(“BlackBackground”, false);

run(“Make Binary”);

run(“Analyze Particles…”, “size=10‐Infinity pixel circularity=0.2‐1.00 display exclude summarize add”);}

For quantification of p‐Tau and amyloid‐β immunohistochemistry staining, imaging was performed using the NanoZoomer scanner (Hamamatsu). The whole section was imaged at 40× magnification. Six representative gray‐matter areas of approximately 1 mm^2^ were analyzed for p‐Tau, and three representative gray‐matter areas of approximately 1 mm^2^ were analyzed for Amyloid‐β. p‐Tau and Amyloid‐β aggregates were quantified manually and blindly using the multi‐point tool in ImageJ (RRID:SCR_002285).

For quantification of 8‐oxoG immunofluorescence staining, imaging was performed using a Leica DM6B microscope at 63× magnification. Signal intensity was measured for 20 cells per sample using ImageJ (RRID:SCR_002285) and normalized to the unaffected control samples.

Data normality was tested using the Anderson‐Darling test. Data that are normally distributed were analyzed using the unpaired *t* test, and data that are not normally distributed were analyzed using the Mann–Whitney *U* test.

### 2.8 Immunoblot analysis of human brain tissue

Frozen human brain tissues from four unaffected control individuals, four GBM patient donors, and three AD patient donors were cut into 40‐μm thick sections. Next, the sections were lysed in 1× Laemmli buffer. After resuspension, the samples were sonicated and centrifuged at 10,621 *g* for 20 min at 4°C. The supernatant was stored at −80°C until use. The total protein concentration was determined using a DC Protein Assay Kit (Bio‐Rad). For protein separation, samples were boiled for 5 min and loaded onto TGX FastCast acrylamide gels 10% (Bio‐Rad).

Proteins were transferred onto nitrocellulose membranes (Bio‐Rad) and blocked using 10% milk powder in PBST. Next, the membranes were incubated overnight at 4°C with specific antibodies against p‐Tau (Ser202, Thr205) (mouse, 1:1000; Thermo Fisher, MN1020, RRID:AB_223647), GAPDH (mouse, 1:10000; Fitzgerald, 10R‐G109A, RRID:AB_1285808), TOMM20 (rabbit, 1:1000; Abcam, ab78547, RRID:AB_2043078), OPA1 (rabbit, 1:1000; Cell Signaling Technology, 80,471, RRID:AB_2734117), MFN1 (rabbit, 1:1000; Cell Signaling Technology, 14,739, RRID:AB_2744531), MFN2 (rabbit, 1:1000; Cell Signaling Technology, 11,925, RRID:AB_2750893), COX‐IV (mouse, 1:1000; Abcam, ab14744, RRID:AB_301443), DRP1 (rabbit, 1:1000; Cell Signaling Technology, 8570 (also 8570S), RRID:AB_10950498), β‐tubulin (rabbit, 1:5000; Sigma‐Aldrich Cat, T2200, RRID:AB_262133), phospho‐Histone H2A.X (mouse, 1:1000; Millipore, 05‐636, RRID:AB_309864), OGG1 (rabbit, 1:1000; Proteintech, 15,125‐1‐AP, RRID:AB_2156780), and β‐tubulin (mouse, 1:20,000; Sigma‐Aldrich, T6074, RRID:AB_477582). Afterwards, the membranes were incubated with anti‐mouse HRP‐linked secondary antibody (1:5000; GE Healthcare, NXA931, RRID:AB_772209), anti‐rabbit HRP‐linked secondary antibody (1:5000; Cell Signaling Technology, 7074 (also 7074S, 7074 V, 7074P2), RRID:AB_2099233), or anti‐rabbit HRP‐linked secondary antibody (1:5000; Bio‐Rad, 170–6515, RRID:AB_11125142). Either Pierce ECL Western Blotting Substrate (Thermo Fisher) or SuperSignal West Dura Substrate (Thermo Fisher) was used for protein visualization. Images for p‐Tau, TOMM20, OPA1, MFN1, MFN2, COX‐IV, DRP1, β‐tubulin and GAPDH were acquired using a ChemiDoc Imaging System (Bio‐Rad) and processed with Image Lab 6.1 software (Bio‐Rad). Images for phospho‐histone H2A.X, OGG1 and β‐tubulin were acquired using the Amersham ImageQuant 800 Imaging System and processed using ImageJ.

Data normality was tested using the Anderson‐Darling test. Data that are normally distributed were analyzed using the unpaired *t* test, and data that are not normally distributed were analyzed using the Mann–Whitney *U* test.

## 3 RESULTS

### 3.1 Transcriptomic analysis of normal‐appearing brain tissue from GBM patients reveals increased levels of inflammation and reduced oxidative phosphorylation

To identify the consequences of a brain tumor and its treatment on the healthy brain, we performed a comparative transcriptomic analysis of human postmortem brain samples derived from healthy subjects and patients with GBM treated with chemotherapy and radiotherapy. We obtained three normal‐appearing brain regions from five GBM patients (NA‐GBM) (two in proximity to the tumor, denoted as “Near #1” and “Near #2” and one further from the tumor, denoted as “Far”), and age‐matched and region‐matched brain tissues from five unaffected control individuals (Figure 1a, Data S1). Survival rates for the GBM patients ranged from 3 months to 4 years after diagnosis (Data S1), and GBM patients suffered from a range of symptoms, including psychiatric, cognitive, sensory, and motor complaints (Data S1, Figure S1).

From these samples, RNA was isolated and bulk RNA‐sequencing was performed. In total, 1298 differentially expressed genes (DEGs) were found in region Near #1 regions, 1208 DEGs were found in region Near #2 regions, and 301 DEGs were found in the Far regions far from the tumor (Figure 1b). This reduction in DEGs with distance from the tumor points to a local impact of the tumor itself and/or radiotherapy (since a higher radiation dose is used closer to the tumor) and less likely the result of a brain‐wide response.

When combining all NA‐GBM and all control samples together and then performing the DEG analysis, 601 upregulated DEGs and 596 downregulated DEGs were detected when comparing the NA‐GBM to unaffected control samples (Figure 1c). The PCA plot showed control and NA‐GBM samples segregated in two clusters, with some overlap (Figure S2a). There was no clustering according to age at time of death, or by brain region (Figure S2b,c). Gene ontology (GO) analysis showed many significantly enriched terms in the upregulated gene clusters. The most significantly enriched terms in the upregulated gene cluster 2 were those involved in inflammation, including regulation of inflammatory response (Figure 1c, Figure S2e). Specifically, the most upregulated pro‐inflammation genes were GBP2 (guanylate binding protein 2), OAS3 (2′‐5′‐oligoadenylate synthetase 3), and IFIT2 and 3 (interferon‐induced protein with tetratricopeptide repeats 2 and 3). Upregulated gene cluster 4 was also enriched for genes involved in protein folding, including HSPB1 (heat shock protein family B (small) member 1), CRYAB (crystallin alpha B), CHORDC1 (cysteine and histidine rich domain containing 1), DNAJB1 (DnaJ heat shock protein family (Hsp40) member B1), and HSPA1A (heat shock protein family A (Hsp70) member 1A). The most significantly enriched terms in downregulated gene clusters 1 and 3 were involved in oxidative phosphorylation and proton transmembrane transport (Figure 1c, Figure S2e). Specifically, the proton transmembrane transport genes most downregulated were NADH subunit NDUFA4, ATP synthase subunits ATP5F1C and ATP5F1B, as well as cytochrome c oxidase subunits COX7B and COX7A2. Cluster 1 also contained an enrichment of downregulated genes involved in the regulation of trans‐synaptic signaling (Figure 1c, Figure S2e). Overall, the GO analysis showed an upregulation of genes involved in inflammation, and a downregulation of genes involved in oxidative phosphorylation.

### 3.2 Gene expression profile of normal‐appearing brain tissue from GBM patients closely resembles Alzheimer's disease

We then asked whether the gene expression changes identified in NA‐GBM brain tissue display an overlap with other disease conditions and compared our DEG dataset with previously published datasets using enrichment analysis against the Molecular Signatures Database. We found a significant number of genes that overlap with the results from an Alzheimer's disease transcriptomic study (Blalock et al., 2004) Alzheimer's disease (AD) transcriptomic study (Figure S2f). The top hit for both the downregulated and the upregulated genes was a publication studying the gene expression in AD patients (Blalock et al., 2004), with a statistically significant overlap of 140 upregulated genes (*p* = 9.06E‐77) and an overlap of 156 downregulated genes (*p* = 3.69E‐111) (Figure S2f).

We observed that Near #1, Near #2 and Far samples shared a similar transcriptomic signature, characterized by upregulation of inflammation and downregulation of oxidative phosphorylation (Figure 1c, Figure S3a,b). Additionally, we repeated the enrichment analysis against the Molecular Signatures Database for the Near #1 region and found similar results in the five most statistically significant hits, with AD as the top hit (Figure 2a). Therefore, we focused on the Near #1 region for further analyses, thereafter referred to as NA‐GBM. The manual comparison between the NA‐GBM DEGs and selected transcriptomes from multiple neurodegenerative diseases (Bottero et al., 2021; Dumitriu et al., 2016; Labadorf et al., 2015; Williams et al., 2021) also showed that AD had the most significant overlap of both up‐ and downregulated genes (Figure 2b). Additionally, parametric gene set enrichment analysis (PGSEA) comparing the NA‐GBM DEGs against the Jensen disease database showed that genes associated with AD positively and significantly correlate with the DEGs we found in NA‐GBM tissue (Figure S3c). Conversely, genes associated with other neurodegenerative diseases (NDs) do not correlate significantly with the DEGs found in NA‐GBM tissue (Figure S3c).

To test the overlap in gene expression between NA‐GBM tissue and AD directly, five additional AD patients were included in our study for differential gene expression analysis. Cell deconvolution analysis confirmed a shared high proportion of excitatory neurons in all samples (Figure S4a). 531 DEGs were upregulated and 631 were downregulated when comparing AD patient samples to unaffected control individual samples (Figure S4b, Data S2). By contrast, only a total of 59 DEGs were differentially regulated when comparing AD and NA‐GBM, suggesting similarities between the two datasets (Figure S4b, Data S2). Comparing the DEGs in the NA‐GBM samples versus control samples and the AD samples versus control samples showed an overlap of 615 DEGs, of which 272 upregulated and 343 downregulated genes (Figure 2c). Overall, there is a significant overlap between the DEGs in NA‐GBM patient samples and the DEGs in AD patient samples (Figure 2d). Unbiased Euclidean clustering confirmed this as the control samples clearly segregated while the AD and NA‐GBM samples were nearly indistinguishable (Figure 2d), in line with the PCA analysis (Figure S4c). Additionally, the PCA plot revealed that sample clustering is not correlated with age (Figure S4d). Together, our findings indicate that the gene expression profile of non‐tumor tissue in GBM patients closely resembles AD.

To evaluate the extent of transcriptomic similarity, the NA‐GBM and AD data sets were combined and compared with the control samples for differential gene expression analysis. We detected 976 upregulated DEGs and 954 downregulated DEGs (Figure 2e, Data S2). GO analysis showed many significantly enriched terms in the upregulated gene clusters involved in different biological processes, the top terms include inflammation, regulation of lipid distribution, regulation of cell death, and extracellular matrix organization (Figure 2e, Figure S4f). Many significantly enriched terms in the downregulated gene clusters are involved in mitochondrial membrane organization, oxidative phosphorylation, and nervous system development, similar to what was observed in the GBM patient analysis (Figure 2e, Figure S4f).

### 3.3 Normal‐appearing brain tissue from GBM patients shows increased DNA damage, oxidative stress and other hallmarks of aging

**FIGURE 3:**
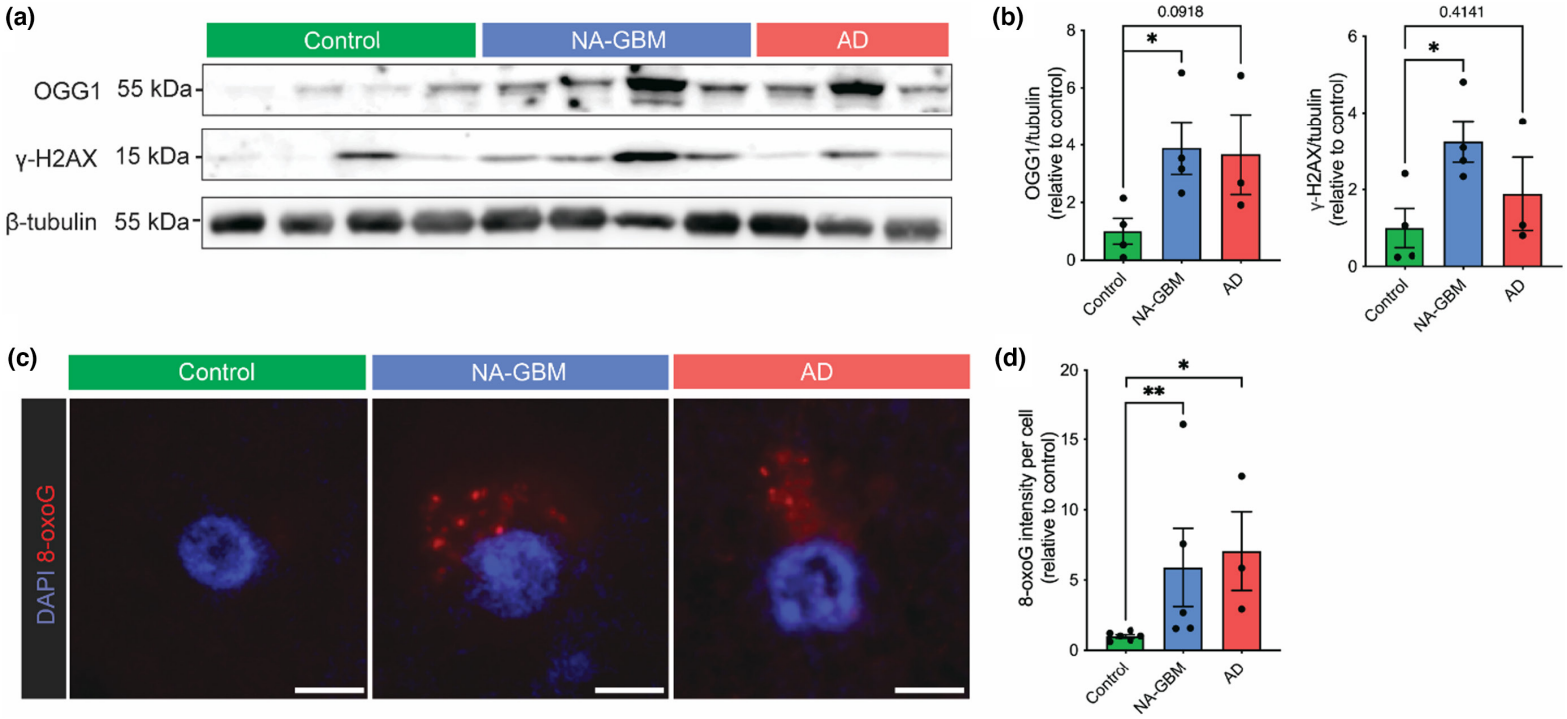
Increase in DNA damage markers in NA‐GBM samples. (a) Western blot analysis of unaffected control, NA‐GBM and AD brain tissue with OGG1, γ‐H2AX, and β‐tubulin antibodies (unaffected control *n* = 4, NA‐GBM *n* = 4, AD *n* = 3). (b) Western blot quantification showing levels of OGG1 relative to β‐tubulin in unaffected control and NA‐GBM brain tissue (*p* = 0.0299 unpaired t test). Western blot quantification showing levels of γ‐H2AX relative to β‐tubulin in unaffected control and NA‐GBM brain tissue (*p* = 0.0233, unpaired t test). Data are represented as mean ± SEM. * indicates a *p* value <0.05. Not significant *p* values are indicated on the individual plot. See Table 1 and Data S1 for further patient information. (c) Representative images of immunofluorescence staining of 8‐oxoG (red) and DAPI (blue) in Control, NA‐GBM and AD samples. Scale bar = 5 μm. (d) Quantification of 8‐oxoG signal intensity per cell in unaffected control, NA‐GBM and AD samples, normalized to 8‐oxoG signal intensity per cell in unaffected control samples (unaffected control *n* = 6, NA‐GBM *n* = 5, AD n = 3). NA‐GBM versus Control *p* value = 0.0043, and AD versus Control *p* value = 0.0238 (Mann–Whitney U). Data are represented as mean ± SEM. * indicates a *p* value <0.05. See Table 1 and Data S1 for further patient information.

Since our RNA‐sequencing analysis indicated mitochondrial dysfunction in the brain of GBM patients, we examined changes in total mitochondrial protein TOMM20 and mitochondrial dynamics‐related proteins using western blot. We observed that while total mitochondrial levels were unaffected in NA‐GBM samples (Figure S5a,b), levels of OPA1 were significantly reduced in NA‐GBM samples (Figure S5a,c). This suggests a reduction in mitochondrial fusion and therefore a loss of mitochondrial maintenance and health, supporting our RNA‐sequencing results that there is a reduction in oxidative phosphorylation (Figure 2e). Other mitochondrial protein levels were unaffected in NA‐GBM samples (Figure S5a,c). In addition, we analyzed markers of oxidative DNA damage by performing western blot for 8‐Oxoguanine glycosylase (OGG1) (Figure 3a,b) and immunofluorescence imaging of 8‐Oxoguanine (8‐oxoG) (Figure 3c,d), both indicating a significant increase in oxidative DNA damage in NA‐GBM samples. We also identified an increase in the double‐strand DNA damage marker γ‐H2AX (Figure 3a,b). Overall, these results are indicative of increased levels of oxidative stress and DNA damage in NA‐GBM samples, comparable to that of AD.

**FIGURE 4:**
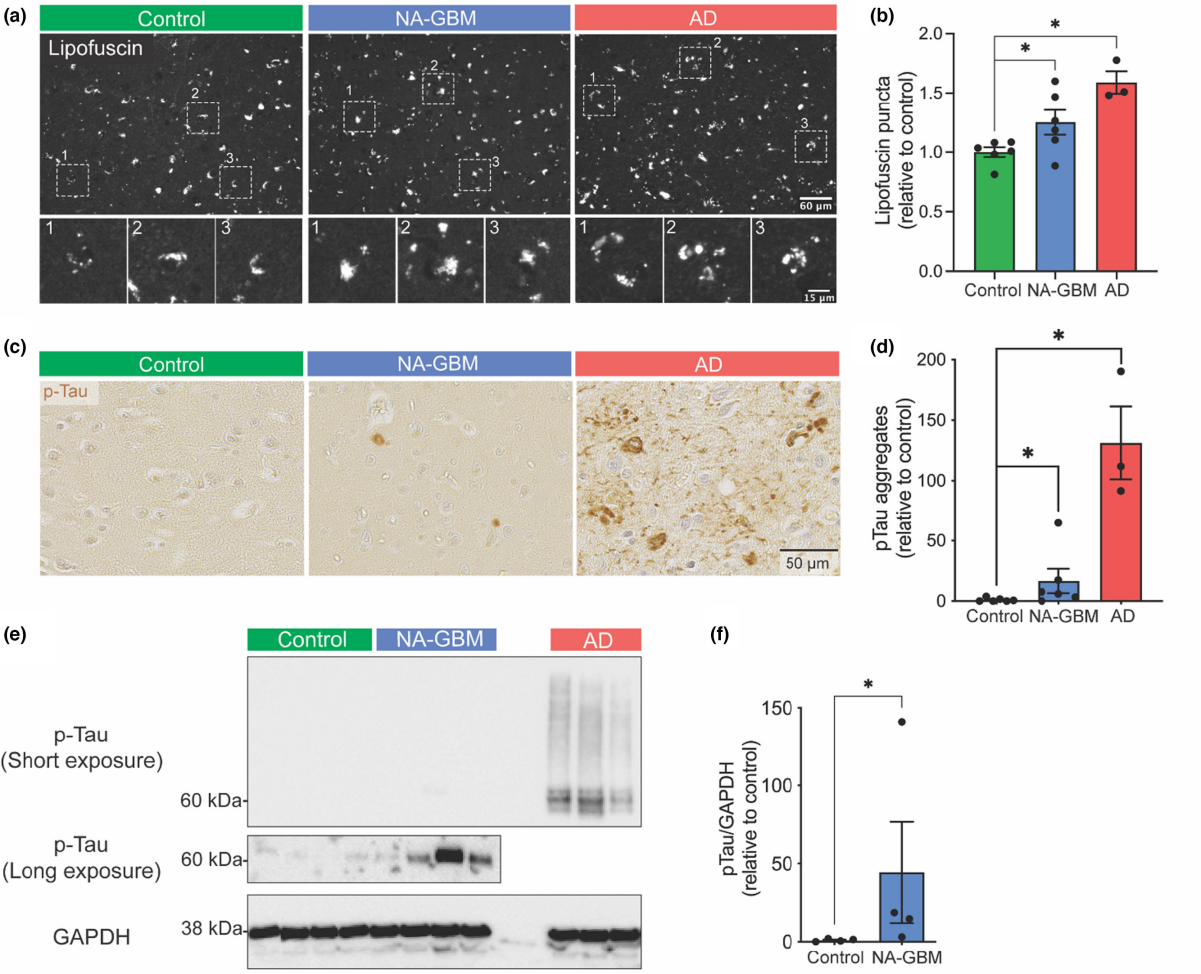
Normal‐appearing GBM patient brain tissue shows increased levels of lipofuscin and hyper‐p‐Tau. (a) Immunofluorescence of lipofuscin granules in unaffected control, NA‐GBM and AD samples. Top panels scale bar = 60 μm. Bottom panels show boxed area in top panels. Bottom panels scale bar = 15 μm. (b) Quantification of number of lipofuscin granules per tile in NA‐GBM and AD samples, relative to the control (Unaffected control *n* = 6, NA‐GBM *n* = 6, AD *n* = 3, NA‐GBM versus unaffected control *p* = 0.0493. AD versus unaffected control *p* = 0.0002, unpaired *t* test. * indicates a *p* value <0.05, *** indicates a *p* value <0.001). Data are represented as mean ± SEM. See Table 1 and Data S1 for further patient information. (c) Immunohistochemistry staining for p‐Tau (Ser202, Thr205) in unaffected control, NA‐GBM and AD samples. Scale bar = 30 μm. (d) Quantification of the p‐Tau IHC staining, showing number of p‐Tau aggregates per mm^2^ in NA‐GBM and AD samples, relative to control samples (unaffected control *n* = 6, GBM *n* = 6, AD *n* = 3. NA‐GBM versus unaffected control *p* = 0.0433. AD versus unaffected control *p* = 0.0238, Mann–Whitney U). Data are represented as mean ± SEM. See Table 1 and Data S1 for further patient information. (e) Western blot analysis of unaffected control, NA‐GBM and AD brain tissue with phosphorylated‐Tau (Ser202, Thr205) antibody and GAPDH antibody (short exposure = 5 s, long exposure = 355 s; unaffected control *n* = 4, NA‐GBM *n* = 4, AD *n* = 3). (f) Western blot quantification showing levels of phosphorylated‐Tau relative to GAPDH in unaffected control and NA‐GBM brain tissue (*p* = 0.0286, Mann Whitney U). Data are represented as mean ± SEM. * indicates a *p* value <0.05. See Table 1 and Data S1 for further patient information.

To further examine the similarity between GBM and AD patient brains at the protein level, we analyzed lysosomal lipofuscin. Accumulation of lipofuscin is a hallmark of aging and age‐related neurodegeneration, including AD (Moreno‐Garcia et al., 2018). There was a significant (*p* = 0.0411) increase in lipofuscin in NA‐GBM patient brain samples compared to control samples (Figure 4a,b). We also observed a significant increase in p‐Tau (Ser202, Thr205, p‐Tau) in NA‐GBM patient samples compared to unaffected control individual samples (Figure 4c,d) (*p* = 0.0433). Tau is a microtubule‐associated protein that becomes hyperphosphorylated and forms insoluble aggregates in neurodegenerative tauopathies including AD (Iqbal et al., 2010). To further confirm that total p‐Tau levels increase in NA‐GBM samples we performed western blot analysis and found a significant increase (*p* = 0.0286) in p‐Tau in NA‐GBM samples compared to control samples, as well as an expected increase of p‐Tau in the AD samples (Figure 4e,f). In contrast, we found no significant increase in Amyloid‐β_42_, another well‐established hallmark of AD, in NA‐GBM samples compared to control samples (Figure S6a,b). In summary, beyond the transcriptional similarities these data suggest that the brain of GBM patients contains hallmarks of accelerated aging and AD‐like neuropathological features.

## 4 DISCUSSION

Our findings reveal that non‐tumor brain regions of GBM patients display hallmarks of accelerated aging. The observed transcriptional overlap with AD suggests that the brain of GBM patients undergoes a similar biological aging process, characterized by inflammation, mitochondrial dysfunction and mis‐regulated proteostasis, and may explain the cognitive decline associated with cancer and its treatment. In our study, we observed an increase in p‐Tau levels in NA‐GBM samples, but absence of amyloid‐β aggregates (Figure 4c,d, Figure S6a,b). p‐Tau forms aggregates in a range of brain pathologies, including AD, progressive supranuclear palsy and corticobasal degeneration (Noble et al., 2013). Therefore, an increase in p‐Tau suggests several possible causes of accelerated aging in the brain of GBM patients, which may differ from AD.

A meta‐analysis‐based transcriptional overlap with AD has also been found in the GBM tumor itself (Sanchez‐Valle et al., 2017) and the presence of hyper‐p‐Tau has been shown in a mouse GBM xenograft model (Lim et al., 2018). These data confirm our findings and hint at the possibility that the AD‐like features might be intrinsic to the tumor cells. Our data now reveal that the overlap with AD is also present in normal‐appearing non‐tumor brain tissue in GBM patients (Figure 2e, Figure S2e). Although we cannot exclude that GBM patients had pre‐existing tauopathies, the gradient in number of DEGs indicates that the present response is unlikely the result of a brain‐wide neurodegenerative pathology, but rather the effect of the tumor and local treatment, such as surgery and radiotherapy.

One of the factors underlying the AD‐like phenotypes observed in GBM patients may be due to the genotoxic effects of radiotherapy and chemotherapy (Carroll et al., 2022; Nonnekens & Hoeijmakers, 2017). Past work in rodents illustrated that cranial irradiation results in behavioral and cognitive changes, and neuroinflammation (Belarbi et al., 2013; Gibson & Monje, 2021; Makale et al., 2017; Montay‐Gruel et al., 2018; Simmons et al., 2019). Compromising DNA repair in AD mouse models also dramatically enhances the similarities with human AD (Sykora et al., 2015). This suggests that DNA‐damaging tumor treatment may be the culprit although further research is necessary to untangle this relationship. In addition, recent studies have shown an association between DNA damage and a loss of protein homeostasis (Huiting et al., 2022; Lee et al., 2021; Schumacher et al., 2021). Remarkably, the proteins that aggregate after genotoxic stress overlap with proteins that aggregate in the background of neurodegenerative diseases including AD (Huiting et al., 2022). DNA damage and reduced expression of DNA damage response proteins have also been implicated in AD (Lin et al., 2020; Sykora et al., 2015). However, how DNA damage can trigger a loss of protein homeostasis remains unclear (Ainslie et al., 2021; Huiting & Bergink, 2021). It has been also previously observed that radiation leads to neuroinflammation (Constanzo et al., 2020), consistent with our observed upregulation of inflammatory genes in GBM patient samples (Figure 1c).

In the present study samples were obtained from relatively younger GBM and AD (average age 56) patients, considering that at diagnosis the median age of a GBM patient is 68–70 years old (Kim et al., 2021), and the average age of an AD patient at presentation is 75 years old (Barnes et al., 2015). Pre‐existing brain aging in older patients could affect the extent of the response observed in the present analysis. Studies using a larger number of GBM patients and matching brain regions of unaffected control individuals are important for further in‐depth analysis and validation of the current findings. Additionally, the present results could be of relevance for pediatric and adult lower grade brain tumors characterized by longer survival rates.

Overall, our study demonstrates that the brain of GBM patients display an AD‐like accelerated aging phenotype. Whether this is due to the impact of the tumor itself, a consequence of radiotherapy and/or chemotherapy treatment or a combination of these remains to be further investigated. The results of this study provide the basis for further testing existing or novel AD therapies, including therapeutic strategies targeting p‐Tau aggregation (Congdon & Sigurdsson, 2018), in brain tumor patients thereby improving their quality of life.

## AUTHOR CONTRIBUTIONS

The study was designed by L.B. and S.B. The paper was written by A.P.A., L.B. and S.B. with input from all authors. The RNA‐sequencing experiments were performed and analyzed by L.B. and A.P.A. The RNA‐sequencing analysis code and further assistance were provided by E.G. Input on gene enrichment analysis and interpretation was provided by B.J.L.E. Lipofuscin stainings were performed and analyzed by A.P.A. p‐Tau stainings were performed and analyzed by M.K. and A.P.A. Amyloid‐β brain stainings were performed and analyzed by L.B. and A.P.A. 8‐oxoG stainings were performed and analyzed by D.C.V and A.P.A. Immunoblots were performed and analyzed by M.K and A.P.A. Neuropathology analysis was performed by W.d.D. Human brain tissues were processed and prepared for analysis by L.B. and D.C.V. Pilot tissue analyses were performed by D.C.V.

## FUNDING INFORMATION

This work was funded by KWF Kankerbestrijding (project numbers 12487 to S.B. and L.B., and 11148 to L.B.).

## CONFLICT OF INTEREST STATEMENT

The authors report no competing interests.

## Supporting information


Figure S1.

Data S1.

Data S2.

## Data Availability

Bulk RNA‐sequencing data are available under GEO number GSE207821. The secure GEO access token is available upon request. Further raw data and analyses are in the supplementary information, or available upon request.
