# Supplementary material for: Glioblastoma and its treatment are associated with extensive accelerated brain aging
Source: Aging Cell. 2024 Jan 17;23(3):e14066. doi: 10.1111/acel.14066 (PMC10928584; doi:10.1111/acel.14066)
Supplement: Supplementary file 1 — Figure S1. [file ACEL-23-e14066-s002.docx]

**Supplementary Figures**

**
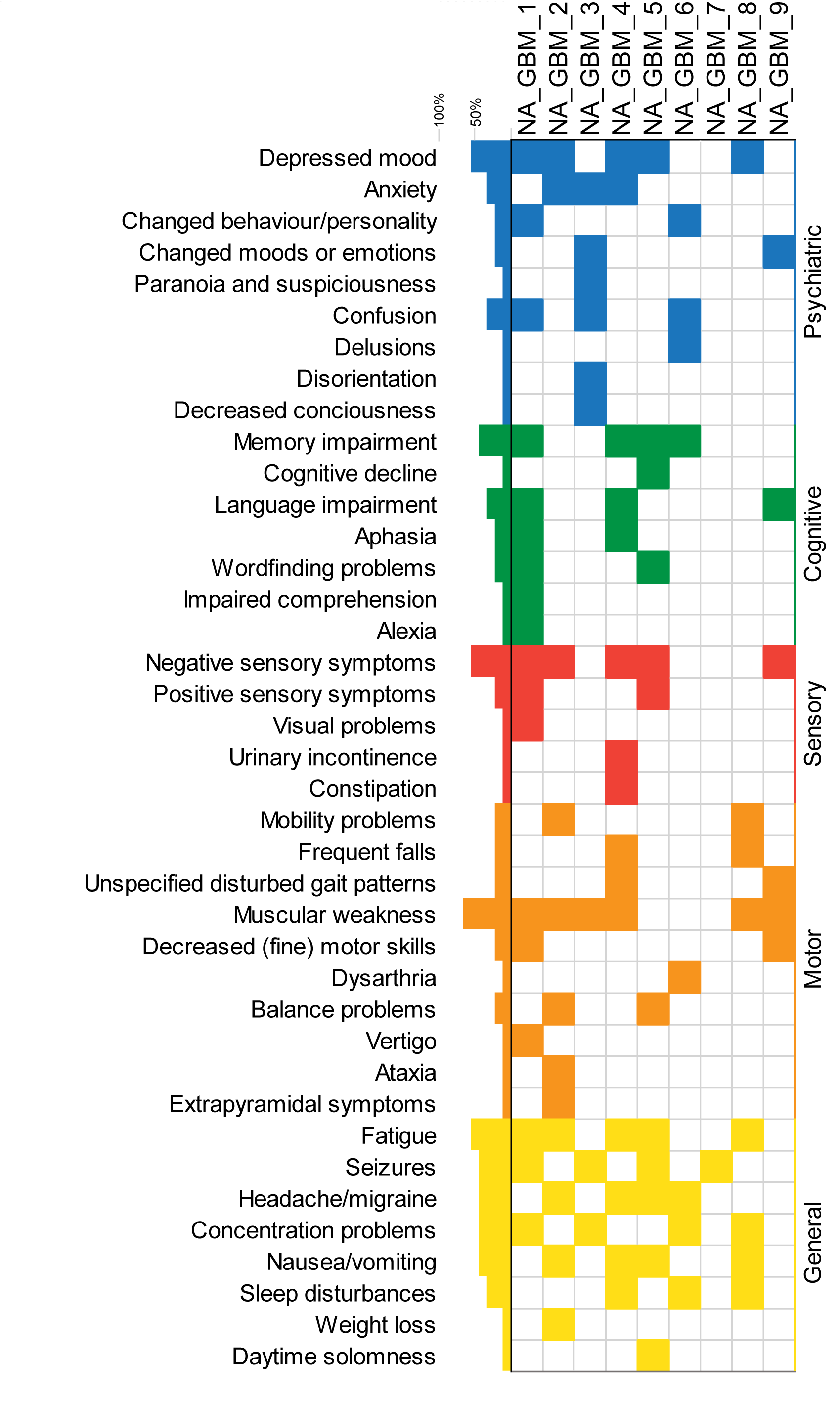
**

**Supplementary Figure 1**

Summary of patient symptoms described in patient files across all 9 GBM patients, categorized into psychiatric, cognitive, sensory, motor and general complaints. The total percentage of patients exhibiting these symptoms is plotted on the left. Further patient information is given in Table 1 and Supplementary Data 1. Symptom categories were adapted from Mekkes *et al* (Mekkes et al., 2022).


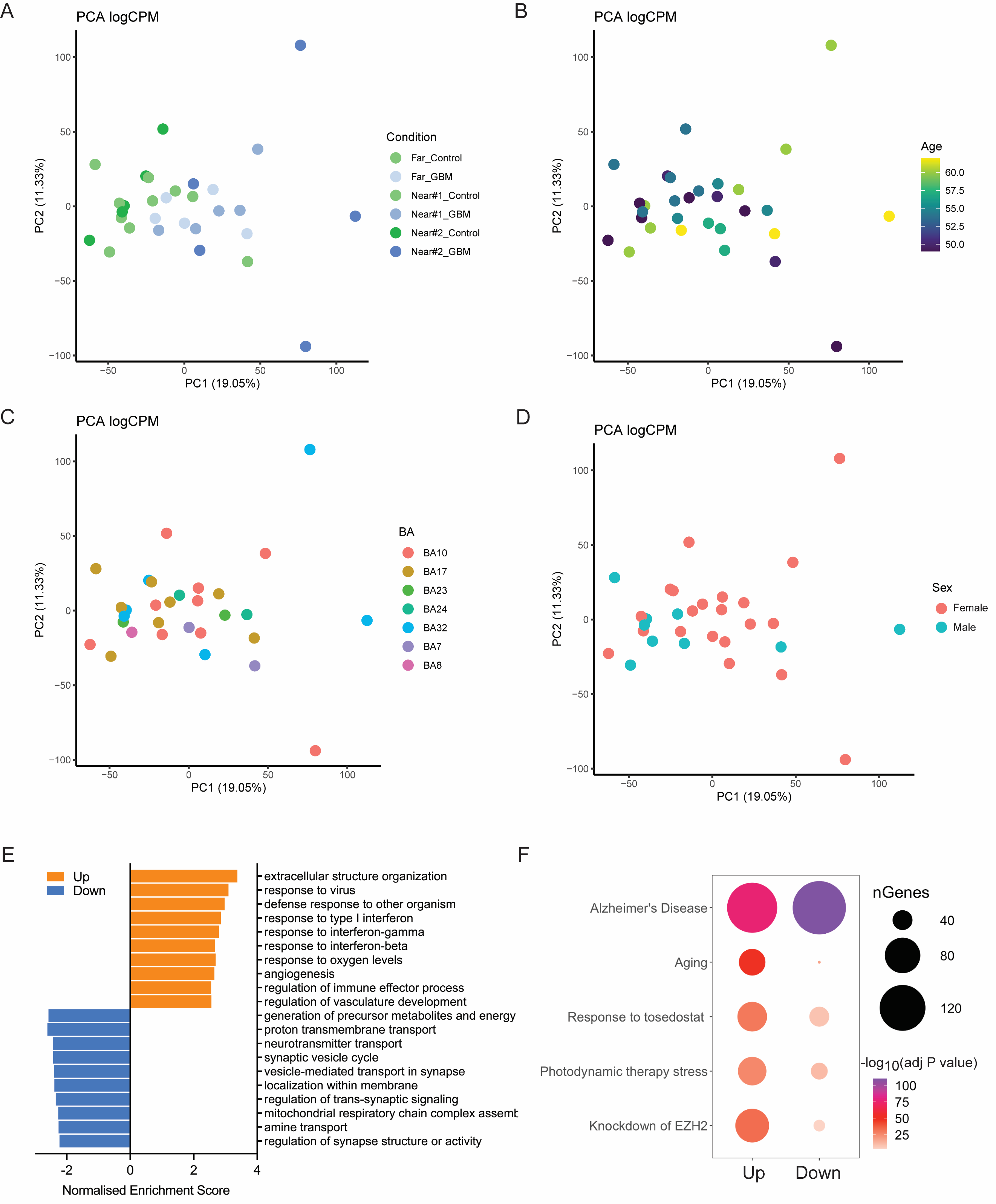


**Supplementary Figure 2**

A-D. Principal component analysis (PCA) plots of NA-GBM patient samples and unaffected control samples, colored by location relative to the tumor (A), age (B), sex (C) and Brodmann Area (D).

E. Biological Processes GO analysis of all NA-GBM brain regions vs region-matched unaffected control brain region DEGs, using WEB-based GEne SeT AnaLysis Toolkit (WebGestalt) (Liao et al., 2019). Orange = upregulated pathway, blue = downregulated pathway.

F. Results of enrichment analysis, comparing all NA-GBM regions vs all unaffected control regions DEG list to the transcriptomic data from the “Chemical and genetic perturbations” data set from the Molecular Signatures Database (MSigDB). The top hits are plotted for the upregulated DEGs and the downregulated DEGs (-log_10_(adjusted P value) > 12). Scale bar indicates -log_10_(adjusted P value) and dot size represents number of overlapping genes.


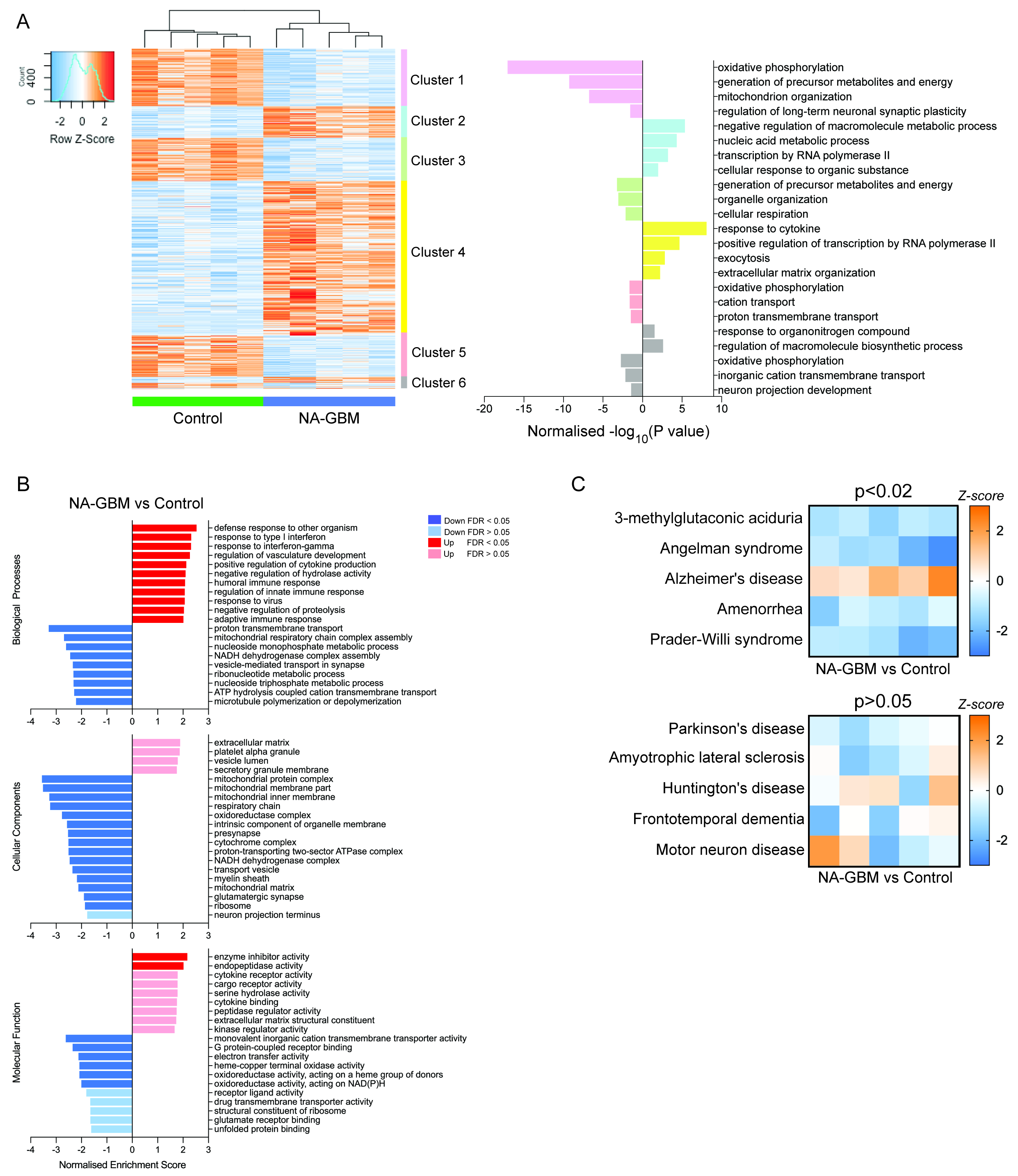


**Supplementary Figure 3**

1. Clustered heat map of DEGs when comparing NA-GBM (Near #1) patient samples to region-matched unaffected controls (fold change > 1.5 and FDR < 0.05). GO analysis (Biological Processes) of individual clusters using g:Profiler, normalized -log_10_(P value) is plotted on the right.
2. GO analysis of NA-GBM (Near #1) vs unaffected control DEGs, Biological Processes, Molecular Functions and Cellular Components, using WEB-based GEne SeT AnaLysis Toolkit (WebGestalt) (Liao et al., 2019). Red = upregulated pathway, blue = downregulated pathway.
3. PGSEA analysis for unaffected control (n = 5) and NA-GBM (Near #1) (n = 5) samples against the Jensen disease database (Grissa, Junge, Oprea, & Jensen, 2022), scale bar indicates the Z-score for each sample. Left plot shows the top 5 hits (p < 0.02), and the right plot shows the results for selected neurodegenerative diseases (p > 0.02).





**Supplementary Figure 4**

**Transcriptomic similarities between NA-GBM and AD patient brain tissue**

1. Cell deconvolution analysis of the unaffected control (n = 5), NA-GBM (Near #1) (n = 5) and AD (n = 5) data sets using the CIBERSORTx analytical tool (Newman et al., 2019). Heat map indicates inferred ratio of cell types. Endo = endothelial, Peri = pericytes, Astro = astrocytes, Oligo = oligodendrocytes, OPC = oligodendrocyte precursor cells, Micro = microglia.
2. Number of DEGs when comparing NA-GBM (Near #1) to control, comparing AD to control, and comparing AD to NA-GBM (Near #1) (fold change > 1.5 and FDR < 0.05).

C-E. Principal component analysis (PCA) plots of NA-GBM patient samples, AD patient samples, and unaffected control samples, colored by Sample type, Post-Mortem Interval (PMI) and Age.

1. GO analysis of NA-GBM (Near #1) and AD vs unaffected control DEGs, Biological Processes, Molecular Functions and Cellular Components, using WEB-based GEne SeT AnaLysis Toolkit (WebGestalt) (Liao et al., 2019). Red = upregulated pathway, blue = downregulated pathway.

**
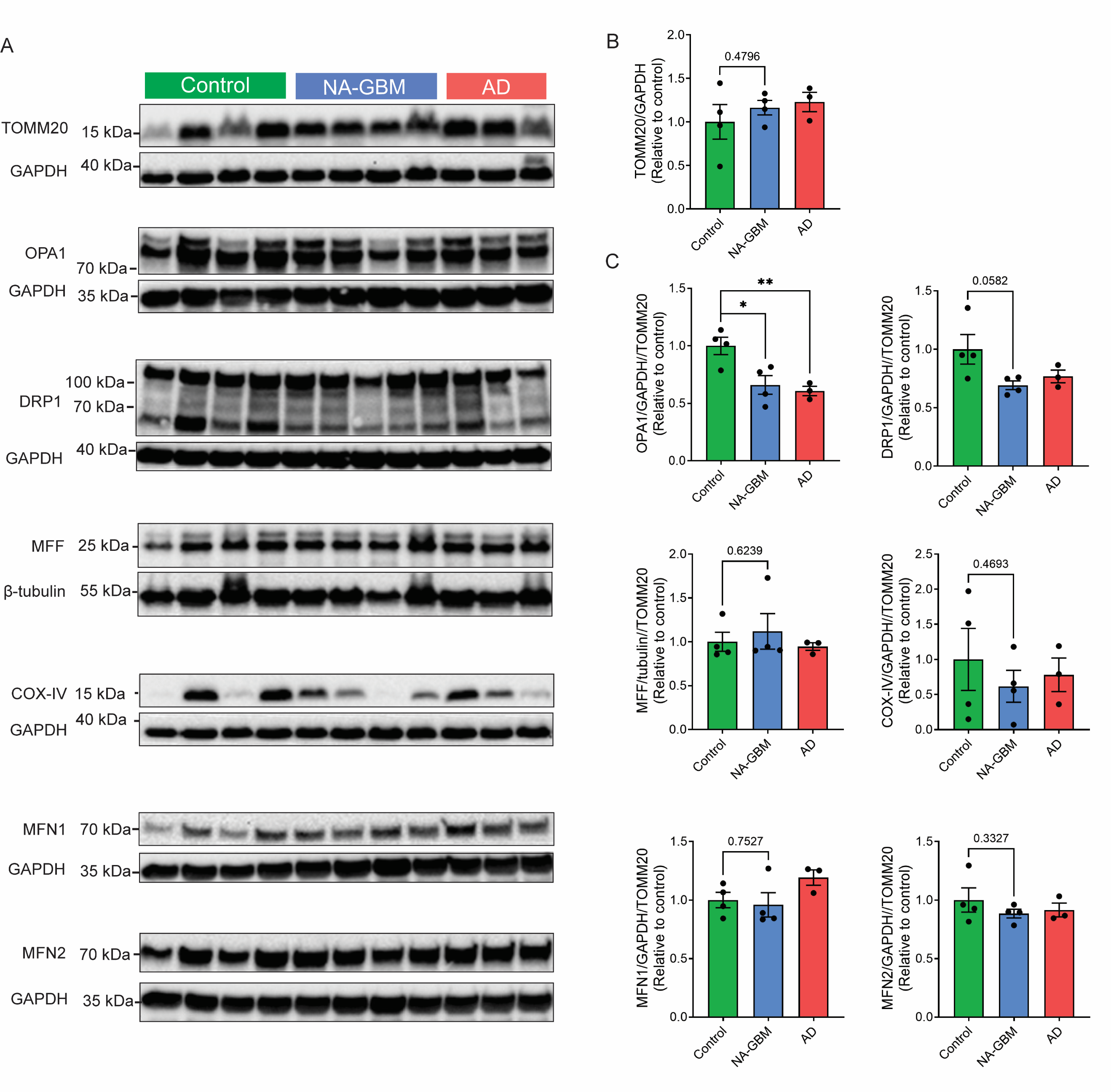
**

**Supplementary Figure 5**

**Decrease in OPA1 mitochondrial fusion protein in NA-GBM patient tissue**

1. Western blot analysis of unaffected control, NA-GBM and AD brain tissue with TOMM20, OPA1, DRP1, MFF, COX-IV, MFN1 or MFN2 antibodies, with β-tubulin or GAPDH antibody as a loading control for each Western Blot.
2. Western blot quantification of TOMM20, relative to loading controls and normalized to unaffected control samples, p = 0.4796 (unpaired t-test).
3. Quantification of Western Blots in Supplementary Figure 5a. Analysis of mitochondrial protein levels in NA-GBM and AD samples, relative to loading controls and relative to TOMM20 levels, and normalized to Unaffected Control samples. P values of not significant comparisons are indicated on each individual plot. OPA1 levels are significantly lower in NA-GBM brain tissue (p = 0.0221 unpaired t-test), and in AD tissue (p=0.0093 unpaired t-test) related to unaffected control samples. * indicates a p value <0.05, ** indicates a p value <0.01. Data are represented as mean ± SEM. See Table 1 and Supplementary Data 1 for further patient information.


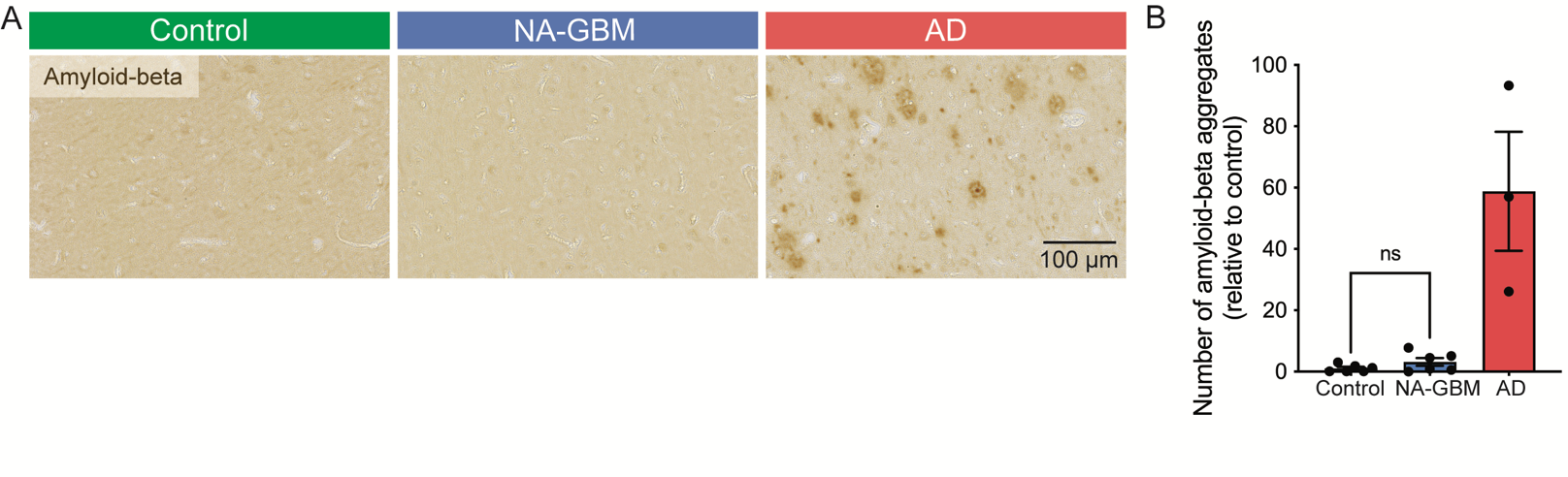


**Supplementary Figure 6**

**NA-GBM samples do not show any increase in amyloid-β levels**

1. Immunohistochemistry staining for amyloid-β in unaffected control, NA-GBM and AD samples. Scale bar = 30 µm.
2. Quantification of the amyloid-β staining, showing number of amyloid-β aggregates per mm^2^ in unaffected control, NA-GBM and AD samples, relative to the controls. Unaffected control n = 6, NA-GBM n = 6, AD n = 3 (GBM vs unaffected control p = 0.2597, AD vs unaffected control p = 0.0238, Mann Whitney U). Data are represented as mean ± SEM. Further patient and sample information is provided in Table 1 and Supplementary Data 1.

**Supplementary Data 1: Description of post-mortem brain tissue samples used in this study**

Table includes sample information about the age at time of death, sex and brain region used of unaffected control individuals, GBM patients and AD patients, and which samples were used for sequencing, immunohistochemistry and western blot analyses. For normal appearing (NA)-GBM patient samples, the approximate tumor location, and details concerning the patient treatment, are also included.

**Supplementary Data 2: DEG lists**

List of DEGs in multiple analyses (fold change > 1.5, FDR < 0.05):

A. All NA-GBM regions vs all unaffected control regions.

B. NA-GBM patient vs unaffected control brain tissue.

C. AD patient vs unaffected control brain tissue.

D. NA-GBM and AD patient vs unaffected control brain tissue.

E. NA-GBM patient vs AD patient brain tissue.

F. Overlapping DEGs between NA-GBM vs unaffected control and AD vs unaffected control.
